# Intraoperative corticosteroid administration for resectable gastric cancer: a multicenter, randomized, open-label, phase II/III study

**DOI:** 10.1007/s10120-025-01635-5

**Published:** 2025-07-10

**Authors:** Takaomi Hagi, Yukinori Kurokawa, Takeshi Omori, Yusuke Akamaru, Keijiro Sugimura, Masaaki Motoori, Jin Matsuyama, Takuro Saito, Kazuyoshi Yamamoto, Tsuyoshi Takahashi, Toshio Shimokawa, Hidetoshi Eguchi, Yuichiro Doki

**Affiliations:** 1 Department of Gastroenterological Surgery, Graduate School of Medicine, The University of Osaka, Osaka, Japan; 2 Department of Gastroenterological Surgery, Osaka International Cancer Institute, Osaka, Japan; 3 Department of Surgery, Osaka Rosai Hospital, Osaka, Japan; 4 Department of Surgery, Kansai Rosai Hospital, Hyogo, Japan; 5 Department of Surgery, Osaka General Medical Center, Osaka, Japan; 6 Department of Gastroenterological Surgery, Higashiosaka City Medical Center, Osaka, Japan; 7 Clinical Study Support Center, Wakayama Medical University, Wakayama, Japan

**Keywords:** Gastric cancer, Corticosteroid, Inflammation, C-reactive protein

## Abstract

**Background:**

Excessive surgical stress induces inflammatory cytokine release, negatively impacting prognosis in patients with malignancies. This study aimed to determine whether the anti-inflammatory effect of a corticosteroid (CS) would improve prognosis when administered intraoperatively to patients with resectable gastric cancer.

**Methods:**

In this multicenter, randomized, open-label, phase II/III study, patients with cStage II–III gastric cancer were randomized to CS administration or non-administration (control) groups. Patients in the CS group received 5 mg/kg of methylprednisolone just before the skin incision during surgery. The primary endpoints were the highest postoperative serum level of C-reactive protein (CRP_max_) in the phase II portion, and recurrence-free survival (RFS) in the phase III portion.

**Results:**

Between December 2016 and February 2019, 410 patients were enrolled. In the phase II portion, CRP_max_ was significantly lower in the CS group than in the control group (mean, 10.7 vs 14.3 mg/dL, respectively; *P* = 0.009). In the phase III portion, 3-year RFS rates in the CS (n = 202) and control (n = 204) groups were 67.2% and 63.0%, respectively, indicating no significant difference (hazard ratio, 0.807 [95% confidence interval, 0.590–1.105]; log-rank *P* = 0.182). Subgroup analysis showed that both histological type and clinical stage had significant interactions with RFS, suggesting a potential survival benefit of CS administration in patients with differentiated histological-type or cStage III gastric cancer.

**Conclusions:**

Intraoperative CS administration mitigated postoperative CRP elevation but did not result in significantly improved survival in patients with cStage II–III gastric cancer. The study is registered with UMIN-CTR, number UMIN000024465.

## Introduction

Gastric cancer remains one of the most common digestive tract malignancies and is the cancer type with the highest incidence and mortality rate in Japan [1]. Gastrectomy is the only curative therapeutic modality and is the first choice in clinical treatment [2, 3]. In recent years, emerging evidence has demonstrated the safety and efficacy of minimally invasive treatments such as laparoscopic and robotic surgery, leading to their widespread use in both Asia and Western countries [4–7]. However, postoperative complications are still unavoidable, with the incidence of severe complications ranging from 3 to 23% [2].

A retrospective study in patients undergoing curative resection for gastric cancer showed that postoperative complications of Clavien–Dindo grade II or higher were an independent prognostic factor [8]. Additionally, we previously demonstrated in a large-scale multicenter study that the postoperative C-reactive protein (CRP) level was a better predictor of prognosis in patients with gastric cancer than the occurrence of intra-abdominal infectious complications [9]. Serum CRP levels are known to correlate with the serum levels of several proinflammatory cytokines, such as interleukin-6 (IL-6) [10], and IL-6 has been reported to induce tumor progression via hyperactivation of the IL-6/JAK/STAT3 pathway [11]. Therefore, elevated postoperative serum IL-6 levels may be associated with prognosis in patients with gastric cancer, and suppression of this cytokine is expected to improve survival.

Glucocorticoids, which are hormones produced and released by the adrenal cortex, function as immunosuppressive and anti-inflammatory agents by inhibiting the release of inflammatory cytokines [12]. Accordingly, we hypothesized that intraoperative corticosteroid (CS) administration would improve the prognosis of gastric cancer. Using a murine model, we previously demonstrated that CS administration inhibited both tumor growth and metastasis induced by systemic inflammation mimicking that caused by surgical stress in humans [13, 14]. However, clinical studies on intraoperative CS administration for gastric cancer have not yet been reported, and its efficacy and safety remain unknown. Here, we conducted a multicenter, randomized, phase II/III study to confirm the survival benefit of intraoperative CS administration in patients with resectable clinical stage II or III gastric cancer. Initially, we examined the efficacy of intraoperative CS administration in terms of reducing postoperative CRP elevation in the phase II portion of the study. Subsequently, we seamlessly transitioned into the phase III portion to evaluate its survival benefit.

## Methods

### Patients

The eligibility criteria of this study were as follows: (1) histologically proven primary gastric cancer; (2) clinical Stage (cStage) II or III (assessed after treatment in patients who underwent neoadjuvant chemotherapy); (3) feasibility of R0 resection; (4) planned total, distal, or proximal gastrectomy; (5) age 20–80 years; (6) eastern cooperative oncology group performance status of 0 or 1; and (7) most recent blood tests within 28 days before registration meeting the following criteria: white blood cell count, 3000–12000/mm^3^; platelet count, ≥ 75000/mm^3^; aspartate aminotransferase and alanine aminotransferase, ≤ 100 IU/L; total bilirubin, ≤ 2.0 mg/dL; and creatinine, ≤ 1.5 mg/dL. The exclusion criteria were active coexisting cancer (simultaneous multiple cancers or multiple cancers with a disease-free interval of less than 5 years, excluding carcinoma in situ or lesions equivalent to intramucosal carcinoma considered cured by local treatment), poorly controlled diabetes or receiving insulin therapy, active infections, continuously administered CS, a history of allergy to CS, a history of myocardial infarction or stroke, and positive for hepatitis B antigen or human immunodeficiency virus antibody. Written informed consent was obtained from all patients before enrollment. Tumors were staged in accordance with 14th version of the Japanese Classification of Gastric Carcinoma (corresponding to the 3rd English edition) [15]. The study protocol was approved by the Institutional Review Board of each participating institution. The study was conducted in accordance with the Helsinki Declaration precepts and was registered with the University Hospital Medical Information Network Clinical Trials Registry (UMIN-CTR) of Japan (identification number UMIN000024465, https://www.umin.ac.jp/ctr/index-j.htm).

### Study design and treatments

This multicenter, randomized, open-label, phase II/III study was conducted by the Clinical Study Group of Osaka University. Patients were randomly assigned (1:1) to either CS administration (CS group) or non-administration (control group), with stratification by institution, cStage (II vs III), planned surgical procedure (total gastrectomy vs distal or proximal gastrectomy), and planned surgical approach (open vs laparoscopic or robotic). The supporting center for clinical research and education (SCCRE) data center assigned the treatment, and no patients or investigators were blinded to the assignment.

Only patients assigned to the CS group received intravenous administration of 5 mg/kg of methylprednisolone sodium succinate just before the skin incision. The protocol did not restrict the use of CS for anaesthesiological reasons such as postoperative nausea and vomiting prophylaxis. Although the type of gastrectomy, surgical approach, degree of lymph node dissection, reconstruction method, and indication for adjuvant chemotherapy were not specified, investigators performed all surgical treatments, at least in principle, in accordance with the 4th edition of the Japanese Gastric Cancer Treatment Guidelines [16].

### Outcomes

The primary endpoint for the phase II portion of the study was the highest postoperative serum level of CRP (CRP_max_) during the hospital stay. The serum levels of CRP were generally measured on day 1, 3, and 7. The CRP_max_ within 30 days after surgery was analyzed instead if the patient was hospitalized for at least 31 days postoperatively. The primary endpoint for the phase III portion was recurrence-free survival (RFS), defined as the period between the date of surgery and the date of recurrence or death by any cause, whichever occurred first. Patients who were alive without recurrence at the time of the analysis were censored at the date of the last confirmation of no recurrence. Secondary endpoints included overall survival (OS), defined as the period between the date of surgery and death from any cause, the postoperative length of hospital stay, the incidence of postoperative complications assessed according to the Clavien–Dindo classification [17, 18], and the postoperative serum level of IL-6. The serum level of IL-6 was measured by a clinical laboratory testing service (SRL, Tokyo, Japan).

### Statistical analysis

For the phase II portion of the study, we assumed according to our preliminary research [19] that the mean CRP_max_ would be 12.8 mg/dL (standard deviation of 6.7 mg/dL) in the control group, and that this value would decrease by an average of 3 mg/dL in the CS group. As such, we calculated that a sample size of 90 patients would provide a one-sided α of 0.1 and a statistical power of 80%. As we anticipated that a few patients would drop out, we planned to enroll a total of 100 patients. These patients were also included in the phase III portion of the study. For the phase III portion, we surmised according to our preliminary research that the 3-year RFS rate would be 73% in the control group, and would improve by 7% in the CS group, and as such we calculated that a sample size of 494 patients would provide a one-sided α of 0.05 and a statistical power of 70%. To compensate for patients dropping out, we planned to enroll a total of 500 patients.

The full analysis set (FAS) for efficacy was defined as all patients enrolled in the study, excluding those with eligibility criteria violations, and an intention-to-treat analysis was conducted. The primary analysis of the phase II portion of the study was conducted upon registration of the first 100 cases. Targeting the FAS, the mean CRP_max_ was calculated and the difference between the CS and control groups was analyzed using the *t*-test (one-sided α = 0.1). If the CS group had a significantly lower CRP_max_ than the control group, phase III proceeded as planned. If no significant difference was observed, the necessity of study termination was reviewed by the Data and Safety Monitoring Committee.

The associations between clinicopathological factors and outcomes in the two groups were examined using Fisher’s exact test or the chi-square test for categorical variables and the Mann–Whitney *U* test for continuous variables. In the primary analysis of the Phase III portion, RFS was estimated with the Kaplan–Meier method and compared using the stratified log-rank test (one-sided α of 0.05) with the clinical stage, surgical procedure, and surgical approach as stratification factors. Adjusted hazard ratios (HRs) and 95% confidence intervals (CIs) for RFS were estimated using the Cox proportional hazards model with stratification factors. To evaluate interactions between background factors and the treatment effect on RFS, post hoc analyses were conducted. The HR for the CS group versus the control group, along with its 95% CI was estimated using an unstratified Cox proportional hazards model for each subgroup and was presented graphically using a forest plot. Statistical analyses were performed using R (version 4.3.0; The R Foundation for Statistical Computing, Vienna, Austria) or the SPSS Statistics software (version 24.0; IBM Corp., Armonk, NY, USA).

## Results

### Patient characteristics

**Fig. 1:**
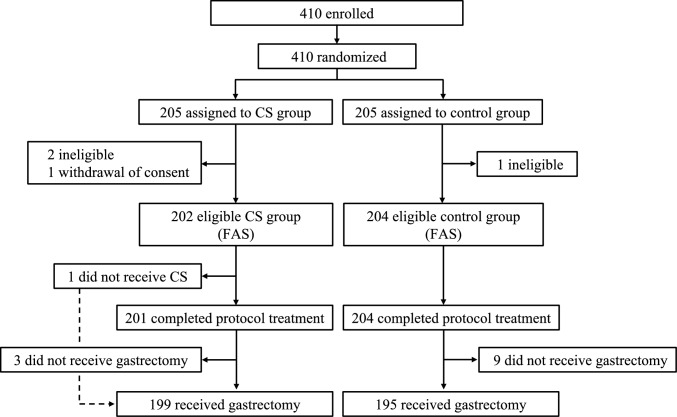
Trial profile

Between December 1, 2016, and February 22, 2019, 410 patients from 23 hospitals in Japan were enrolled and randomly assigned to the CS group (n = 205) or the control group (n = 205). Three patients were judged ineligible, while one patient withdrew consent. The remaining 406 patients (202 CS and 204 control) comprised the FAS dataset (Fig. 1). One patient in the CS group did not receive CS, and was determined to constitute a protocol violation. In addition, three patients in the CS group and nine patients in the control group did not undergo gastrectomy due to peritoneal or hepatic metastasis or invasion to other organs, all of which were identified during surgery.

Baseline characteristics were well balanced between the two groups (Table 1). Of all 406 patients, 222 (54.7%) were cStage II and 184 (45.3%) were cStage III. Total gastrectomy was performed in 118 (29.1%) patients, and open surgery was performed in 194 (47.8%). R0 resection was achieved in 184 (92.5%) patients in the CS group and 175 (89.7%) in the control group.

**Table 1:** Patient characteristics

	CS group (n = 202)	Control group (n = 204)	*P* value
Age (years)			0.31
Median (range)	70 (35 – 80)	69 (35 – 80)	
Sex			0.16
Male	146 (72.3%)	134 (65.7%)	
Female	56 (27.7%)	70 (34.3%)	
ECOG performance status			1.00
0	175 (86.6%)	177 (86.8%)	
1	27 (13.4%)	27 (13.2%)	
Tumor location			0.58
Upper third	49 (24.3%)	58 (28.4%)	
Middle third	76 (37.6%)	69 (33.8%)	
Lower third	77 (38.1%)	77 (37.7%)	
Macroscopic type			0.16
Type 4	27 (13.4%)	18 (8.8%)	
Non-Type 4	175 (86.6%)	186 (91.2%)	
Histological type*			1.00
Differentiated	107 (53.0%)	106 (52.7%)	
Undifferentiated	93 (46.0%)	93 (46.3%)	
Others	2 (1.0%)	2 (1.0%)	
Clinical stage			1.00
II	110 (54.5%)	112 (54.9%)	
III	92 (45.5%)	92 (45.1%)	
Preoperative chemotherapy			0.50
No	167 (82.7%)	174 (85.3%)	
Yes	35 (17.3%)	30 (14.7%)	
Surgical procedure			0.75
Total gastrectomy	63 (31.2%)	55 (27.0%)	
Distal gastrectomy	122 (60.4%)	125 (61.3%)	
Proximal gastrectomy	14 (6.9%)	15 (7.4%)	
No resection	3 (1.5%)	9 (4.4%)	
Surgical approach			0.77
Open	95 (47.0%)	99 (48.5%)	
Laparoscopic/Robotic	107 (53.0%)	105 (51.5%)	
Lymph node dissection			0.58
Less than D2	20 (9.9%)	23 (11.3%)	
D2 or more	179 (88.6%)	172 (84.3%)	
No resection	3 (1.5%)	9 (4.4%)	
Operation time (min)			0.92
Median (range)	259 (56 – 699)	273 (34 – 556)	
Blood loss (mL)			0.93
Median (range)	100 (0 – 1915)	100 (0 – 1965)	
Pathological T status^†^			0.50
T0	1 (0.5%)	1 (0.5%)	
T1	24 (12.6%)	25 (12.8%)	
T2	37 (18.6%)	28 (14.4%)	
T3	68 (34.2%)	70 (35.9%)	
T4a	66 (33.2%)	62 (31.8%)	
T4b	3 (1.5%)	9 (4.6%)	
Pathological N status^†^			0.45
N0	71 (35.7%)	66 (33.8%)	
N1	36 (18.1%)	31 (15.9%)	
N2	30 (15.1%)	44 (22.6%)	
N3a	40 (20.1%)	35 (17.9%)	
N3b	22 (11.1%)	19 (9.7%)	
Pathological stage^†^			0.83
I	43 (21.6%)	36 (18.5%)	
II	60 (30.2%)	61 (31.3%)	
III	82 (412%)	81 (41.5%)	
IV	14 (7.0%)	17 (8.7%)	
Residual tumor^†^			0.45
R0	184 (92.5%)	175 (89.7%)	
R1	13 (6.5%)	15 (7.7%)	
R2	2 (1.0%)	5 (2.6%)	

Clinical and pathological stage was according to the 14th Japanese Classification of Gastric Carcinoma

^*^Data on histological type were not available for three patients

^†^Twelve patients (three in the CS group and nine in the control group) who did not undergo gastrectomy were excluded

*CS* corticosteroids, *ECOG* eastern cooperative oncology group

### Postoperative outcomes

The last of the 100 eligible patients was enrolled on September 30, 2017, making the completion of patient enrollment for the phase II portion of the study. Excluding four patients who did not undergo gastrectomy, the remaining 50 CS and 46 control patients were included in the analysis. The mean CRP_max_ was significantly lower in the CS group compared with the control group (10.7 vs 14.3 mg/dL, respectively; one-sided *P* = 0.009), leading us to proceed to the phase III portion as planned.

**Fig. 2:**
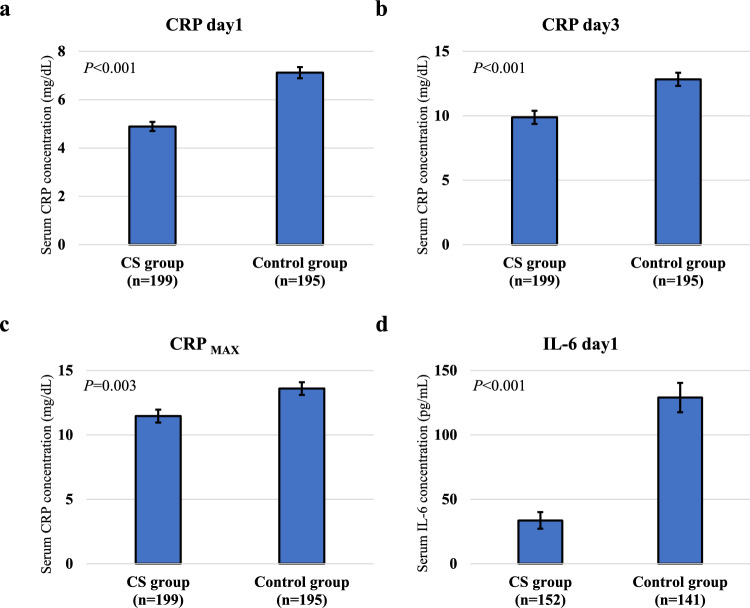
Postoperative serum levels of C-reactive protein (CRP) and interleukin-6 (IL-6). Serum levels of CRP on postoperative **a** day 1 and **b** day 3, and **c** the highest postoperative level (CRP_max_). **d** Serum levels of IL-6 on postoperative day 1. Data are means ± standard error

Among 394 patients who were included in the FAS dataset but excluding the 12 who were not analyzed because they did not undergo gastrectomy, those in the CS group compared with the control group showed significantly lower serum CRP levels on postoperative day 1 (mean, 4.89 vs 7.12 mg/dL, respectively; *P* < 0.001) and day 3 (mean, 9.88 vs 12.83 mg/dL, respectively; *P* < 0.001), and also a significantly lower CRP_max_ (mean, 11.46 vs 13.60 mg/dL, respectively; *P* = 0.003) (Fig. 2). Additionally, among 293 patients for whom the serum IL-6 level was measured on postoperative day 1, the level was significantly lower in the CS group than in the control group (mean, 33.6 vs 129.0 pg/mL, respectively; *P* < 0.001).

The safety analysis included 198 patients in the CS group who had no protocol violations, 195 patients in the control group who underwent gastrectomy, but excluded one patient in the CS group who had a protocol violation. No significant difference in the overall incidence of complications of grade II or higher was observed between the two groups: 37 (18.7%) in the CS group and 33 (16.9%) in the control group (*P* = 0.69) (Table 2). The postoperative length of hospital stay was similar between the two groups (*P* = 0.28), with a median of 11 days (range 6–69) in the CS group and 12 days (range 5–115) in the control group. No hospital death occurred in either group.

**Table 2:** Postoperative details

	CS group (n = 198)	Control group (n = 195)	*P* value
Any complications (C-D grade ≥ II)	37 (18.7%)	33 (16.9%)	0.69
Pancreatic fistula	10 (5.1%)	12 (6.2%)	
Anastomotic leak	11 (5.6%)	3 (1.5%)	
Intraabdominal abscess	4 (2.0%)	7 (3.6%)	
Pneumonia	2 (1.0%)	6 (3.1%)	
Postoperative hemorrhage	3 (1.5%)	3 (1.5%)	
Wound infection	2 (1.0%)	4 (2.1%)	
Intestinal obstruction	3 (1.5%)	1 (0.5%)	
Anastomotic stenosis	1 (0.5%)	0 (0%)	
Mediastinitis	1 (0.5%)	0 (0%)	
Stroke	0 (0%)	1 (0.5%)	
Others	4 (2.0%)	5 (2.6%)	
Postoperative length of stay (days)			0.28
Median	11	12	
Range	6 – 69	5 – 115	

*CS* corticosteroids, *C-D* Clavien-Dindo classification

### Survival rates

**Fig. 3:**
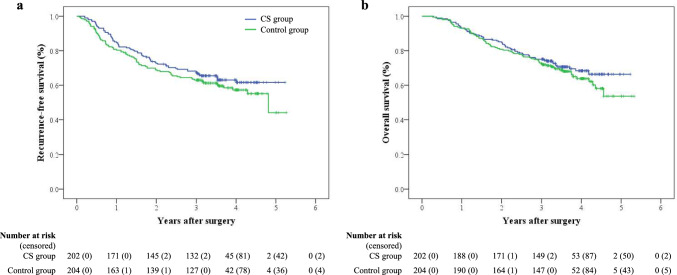
Kaplan–Meier survival curves of **a** recurrence-free survival and **b** overall survival in all randomly assigned patients

The median follow-up period for all censored patients in the FAS dataset was 3.6 years. RFS events were observed in 73 of 202 patients (36.1%) in the CS group and 84 of 204 patients (41.2%) in the control group during the follow-up period. The 3-year RFS rate was 67.2% in the CS group and 63.0% in the control group (adjusted HR, 0.807 [95% CI 0.590–1.105]; stratified log-rank *P* = 0.18) (Fig. 3a). Deaths from any cause during the follow-up period were reported in 60 patients (29.7%) in the CS group and 71 (34.8%) in the control group. The 3-year OS rate was 75.1% in the CS group and 72.5% in the control group (HR, 0.847 [95% CI 0.600–1.195]; log-rank *P* = 0.34) (Fig. 3b). Of 406 patients, 64 (31.7%) in the CS group and 74 (36.3%) in the control group had gastric cancer recurrence. The most frequent site of recurrence was the peritoneum in both groups (CS 16.3%; control, 16.7%), followed by the lymph nodes (CS 5.0%; control, 8.3%) and liver (CS 5.0%; control, 6.4%).

**Fig. 4:**
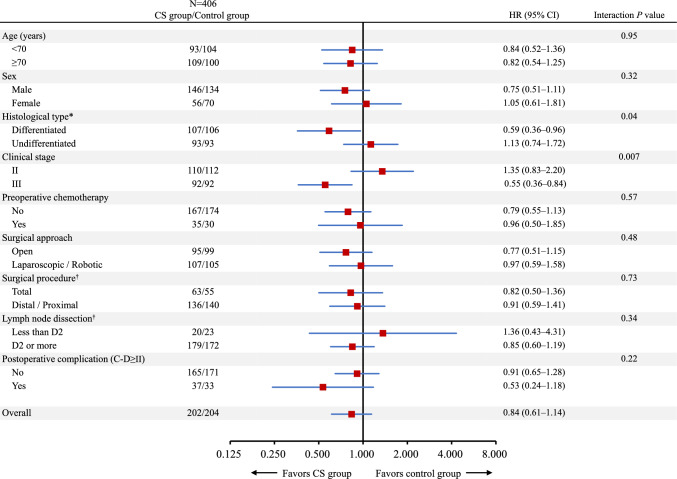
Subgroup analyses of recurrence-free survival (RFS). The forest plot presents hazard ratios (HRs) with 95% confidence intervals (CIs, blue bars) for RFS events in patients assigned to the corticosteroid (CS) group (red squares). *Four patients with neuroendocrine carcinoma and three patients whose histological type was unavailable were excluded. ^†^Twelve patients who did not undergo gastrectomy were excluded

In a subgroup analysis, histological type (interaction *P* = 0.04) and clinical stage (interaction *P* = 0.007) showed significant interactions with RFS (Fig. 4). Among patients with the differentiated type of gastric cancer, the CS group had significantly better RFS than the control group (HR, 0.59 [95% CI 0.36–0.96]; log-rank *P* = 0.03), while the two groups had similar RFS among patients with the undifferentiated type (HR, 1.13 [95% CI 0.74–1.72]; log-rank *P* = 0.58). Similarly, patients with cStage III gastric cancer demonstrated a significant survival benefit in the CS group compared with the control group (HR, 0.55 [95% CI 0.36–0.84]; log-rank *P* = 0.005), whereas patients with cStage II gastric cancer did not (HR, 1.35 [95% CI 0.83–2.20]; log-rank *P* = 0.23).

## Discussion

This multicenter, randomized, phase II/III study demonstrated that intraoperative administration of CS significantly reduced postoperative elevations of serum CRP and IL-6 levels after gastrectomy, without increasing the incidence of postoperative complications. Nevertheless, CS administration did not significantly prolong RFS after surgery for cStage II or III gastric cancer. However, among patients with differentiated histological-type or cStage III gastric cancer, the CS group had significantly better RFS than the control group. The survival benefit of CS in these subgroups should be reexamined in a future trial.

In Japan, intraoperative administration of CS is common during esophagectomy for esophageal cancer, and it has been weakly recommended by Japanese esophageal cancer practice guidelines since 2012 [20, 21]. It has been shown to reduce respiratory failure and in-hospital mortality rates, shorten hospital stays, and suppress postoperative CRP and cytokine elevations without increasing postoperative infectious complications [22, 23]. However, clinical studies on the intraoperative administration of CS for gastric cancer have not yet been reported, and its efficacy and safety remain unknown. A multicenter phase III trial (CORTIFRENCH trial) to assess the impact of intraoperative CS on both short- and long-term outcomes in digestive cancer was recently initiated [24]. The potential efficacy of intraoperative CS administration for various types of cancer is of considerable interest, and the current work can be considered a pioneering study with valuable findings.

We selected CRP_max_ as the primary endpoint for the phase II portion of the study, because our previous large-scale cohort study demonstrated that the postoperative CRP level was a good predictor of prognosis in patients with gastric cancer [9]. We considered CRP_max_ to be a more comprehensive marker of postoperative inflammation, as it captures not only the degree of surgical stress but also the additional inflammatory burden associated with postoperative complications, compared to CRP on postoperative day 1, day 3, or serum IL-6 on postoperative day 1. However, although intraoperative CS administration significantly decreased CRP_max_, there was no significant improvement in prognosis. One possible reason for the negative effect of intraoperative CS administration on survival is that the extent of systemic inflammation in the two groups did not differ as significantly as expected. In our previous study, the difference in the median CRP_max_ between the low and high CRP_max_ groups was 9.54 mg/dL (7.95 mg/dL and 17.49 mg/dL, respectively), while in the present study it was 2.42 mg/dL (CS group, 9.78 mg/dL; control group, 12.2 mg/dL). This discrepancy may be due to an insufficient dose of CS in this study. In previous studies investigating the efficacy of intraoperative CS administration in gastrointestinal surgery, the flash dose of methylprednisolone generally ranged from 10 mg/kg to 30 mg/kg [22, 25, 26], which was more than double the dose used in the present study.

Additionally, the extent of systemic inflammation after gastrectomy may not be large enough for CS to achieve significant benefits. With recent advances in minimally invasive surgery and improvements in surgical techniques, postoperative inflammatory responses may have decreased compared to those observed in patients from our previous retrospective study [9]. Indeed, the subgroup consisting of cStage III patients, who were assumed to undergo relatively more invasive surgery than cStage II patients, showed a survival benefit from intraoperative CS administration. Moreover, patients who experienced postoperative complications, underwent open surgery, or received lymph node dissection of D2 or greater also showed a trend toward prolonged survival after intraoperative CS administration, although the effect was not statistically significant. Even when postoperative complications occur, intraoperative CS administration may suppress the release of cytokines and help prevent deterioration of long-term outcomes. These findings suggest that certain subgroups may potentially derive greater benefit from intraoperative CS administration, and further studies with larger sample sizes focusing on these subpopulations are warranted to validate this possibility.

Our subgroup analysis showed a significant survival benefit of CS administration among patients with the differentiated histological type of gastric cancer. This is probably because patterns of metastasis differ by histological type, *i.e.*, the differentiated histological type is associated with hematogenous metastasis, while the undifferentiated histological type is associated with peritoneal metastasis [27]. Furthermore, CS administered into the peripheral blood is readily transferred to the lymphatic vessels, but less frequently to the peritoneum [28]. Our preclinical study revealed that CS administration reduced the incidence of liver metastases by suppressing the expression of E-selectin in the vascular endothelium of the portal vein [13]. Indeed, in the current study, the difference in the gastric cancer recurrence rate between the CS and control groups was larger in the lymph nodes (5.0 vs 8.3%, respectively) and liver (5.0 vs 6.4%, respectively) than in the peritoneum (16.3 vs 16.7%, respectively).

A negative aspect of steroid use is that it may promote tumorigenesis. A previous study reported that CS reduced T-cell activation by interfering with T-cell receptor signaling [29]. Additionally, CS influences the polarization of T helper (Th) cells, favoring the differentiation of regulatory T cells over that of Th1 cells, which leads to tumor promotion or metastasis [30, 31]. It is unclear whether an intraoperative flash dose of CS causes subsequent persistent changes in tumor immunity, but the aforementioned effects may have influenced our results. In future studies, the use of anti-inflammatory drugs targeting more specific cytokines, such as anti–IL-6 receptor antibodies, may be considered.

Another drawback of steroid use is the potential for side effects. However, no significant difference in the incidence of total postoperative complications or infectious complications was observed between the CS group and the control group in this study. Two systematic reviews and meta-analyses revealed that intraoperative CS administration decreased total complications in hepatic resection, whereas in colorectal surgery there was no difference in total complications, infectious complications, or anastomotic leakage [25, 32]. Since gastrectomy is more similar to colorectal surgery than to hepatic resection in terms of the extent of postoperative inflammation, we believe our results are consistent with those of previous studies. CS administration may have several side effects; among these, hyperglycemia is associated with a higher risk of anastomotic leakage and surgical site infections [33]. Indeed, our study showed a higher incidence of anastomotic leakage, although it was not significant (*P* = 0.062). However, in a nationwide database, preoperative prophylactic use of CS following esophagectomy did not increase anastomotic leakage [22]. Thus, this issue remains controversial and should be further investigated in future studies. Nevertheless, our findings suggest that intraoperative CS administration is safe and does not increase the incidence of steroid-associated side effects.

This study has several limitations. First and foremost, only 82% of the planned number of patients were enrolled. Therefore, the study was insufficiently powered to permit definitive conclusions about long-term outcomes. Second, this was an open-label randomized controlled trial, and the investigators were not blinded to the clinical data or postoperative complications. Third, we did not restrict the use of CS for anaesthesiological reasons such as postoperative nausea and vomiting prophylaxis. Indeed, six patients in the CS group and 21 in the control group received CS outside of protocol treatment for anaesthesiological reasons. However, the results of a sensitivity analysis conducted without these 27 patients were essentially unchanged (data not shown).

In conclusion, intraoperative administration of 5 mg/kg of CS mitigated postoperative CRP elevation; however, it did not provide a significant survival benefit after surgery for cStage II or III gastric cancer.
